# The socioeconomic epidemiology of inherited retinal diseases in Portugal

**DOI:** 10.1186/s13023-024-03161-6

**Published:** 2024-04-09

**Authors:** Ana Marta, João Pedro Marques, Cristina Santos, Luísa Coutinho-Santos, Sara Vaz-Pereira, José Costa, Pedro Arede, Raquel Félix, Sara Geada, Nuno Gouveia, Rui Silva, Margarida Baptista, Miguel Lume, Ricardo Parreira, Célia Azevedo Soares, Maria João Menéres, Carolina Lemos, João Melo Beirão

**Affiliations:** 1 Department of Ophthalmology, Centro Hospitalar Universitário de Santo António, EPE (CHUdSA), Porto, Portugal; 2 Instituto Ciências Biomédicas Abel Salazar (ICBAS), Porto, Portugal; 3 Centro de Responsabilidade Integrado de Oftalmologia do Centro Hospitalar e Universitário de Coimbra, EPE (CRIO-CHUC), Coimbra, Portugal; 4 Clinical Academic Center of Coimbra, Coimbra, Portugal; 5 Faculty of Medicine, University Clinic of Ophthalmology, University of Coimbra (FMUC), Coimbra, Portugal; 6 Instituto de Oftalmologia Dr. Gama Pinto (IOGP), Lisboa, Portugal; 7 Faculdade de Ciências Médicas, NMS, FCM, NOVA Medical School, Universidade NOVA de Lisboa, 7 iNOVA4Health, Lisboa, Portugal; 8 Department of Ophthalmology, Centro Hospitalar Universitário de Lisboa Norte (CHULN), Lisboa, Portugal; 9 Department of Ophthalmology, Faculdade de Medicina, Universidade de Lisboa, Lisbon, Portugal; 10 Department of Ophthalmology, Hospital de Braga (HB), Braga, Portugal; 11 Department of Ophthalmology, Centro Hospitalar Lisboa Ocidental, EPE (CHLO), Lisboa, Portugal; 12 Medical Genetics Department, Centro de Genética Médica Jacinto Magalhães, Centro Hospitalar Universitário de Santo António, EPE (CHUdSA), Porto, Portugal; 13 Unit for Multidisciplinary Research in Biomedicine, Instituto de Ciências Biomédicas Abel Salazar, Universidade do Porto, Porto, Portugal; 14 Medical Science Department, Universidade de Aveiro, Aveiro, Portugal; 15 Instituto de Investigação e Inovação em Saúde, Universidade do Porto, Porto, Portugal; 16 Centro Hospitalar Universitário de Santo António, EPE (CHUdSA), Largo do Prof. Abel Salazar, 4099-001 Porto, Portugal

**Keywords:** Inherited retinal diseases, Epidemiology, Economics, Social

## Abstract

**Background:**

Inherited retinal diseases (IRDs) are a group of rare degenerative disorders of the retina that can lead to blindness from birth to late middle age. Knowing the target population and its resources is essential to better plan support measures. The aim of this study was to evaluate the socioeconomic characteristics of regions in Portugal where IRD patients reside to inform the planning of vision aid and rehabilitation intervention measures.

**Results:**

This study included 1082 patients from 973 families, aged 3 to 92 years, with a mean age of 44.8 ± 18.1 years. Patients living with an IRD were identified in 190 of the 308 municipalities. According to this study, the estimated IRD prevalence in Portugal was 10.4 per 100,000 inhabitants, and by municipalities, it ranged from 0 to 131.2 per 100,000 inhabitants. Overall, regions with a higher prevalence of IRD have a lower population density (*r*=-0.371, *p* < 0.001), a higher illiteracy rate (*r* = 0.404, *p* < 0.001) and an overall older population (*r* = 0.475, *p* < 0.001). Additionally, there is a lower proportion of doctor per capita (*r* = 0.350, *p* < 0.001), higher social security pensions beneficiaries (*r* = 0.439, *p* < 0.001), worse water quality for human consumption (*r*=-0.194, *p* = 0.008), fewer audiences at the cinema (*r*=-0.315, *p* < 0.001) and lower proportion of foreign guests in tourist accommodations (*r*=-0.287, *p* < 0.001).

**Conclusion:**

The number of identified patients with IRD varied between regions. Using data from national statistics (PORDATA), we observed differences in socioeconomic characteristics between regions. Multiple targeted aid strategies can be developed to ensure that all IRD patients are granted full clinical and socioeconomic support.

## Background

Inherited retinal diseases (IRDs) are a group of rare degenerative disorders of the retina, with clinical and genetic heterogeneity that can lead to blindness from birth to late middle age [1, 2]. Economic costs and reduction in the quality of life in IRD are significant and have already been described in some countries [3–6]. In Portugal, a recent study evaluated the visual impairment of IRD patients as per the national table of disability and found that these patients experience significant visual disabilities, with the majority being eligible for a “multipurpose disability medical certificate.“ [7].

Visual impairment is common in Portugal and many people are still struggling to cope with the condition [8]. These individuals frequently used informal care [9]. At the level of education, special education teachers and other professionals with specific training in schools are placed in Portuguese schools by the Ministry of Education to teach specific curriculum areas, such as Portuguese Sign Language, Braille or the use of Support Technologies [10]. Additional visual rehabilitation interventions, alongside usual eye care, may reduce the economic burden of visual loss at personal and societal levels [9]. In Portugal, data about access to low-vision aids are not known, but some barriers could be similar to other countries, such as a lack of awareness [11–13]. 

The epidemiologic landscape of IRDs is critical as they vary considerably between regions and ethnic groups [14, 15]. This knowledge can allow the identification of possible gaps and avenues for intervention across the country including awareness campaigns and the correct management of these patients.

Epidemiological studies of IRD in Portugal are scarce [16, 17]. The creation of a national database helped to identify those patients, and several publications resulted of this joint multicentre collaboration in Portugal [18–21]. Two studies showed clinical characterization of Retinitis Pigmentosa and Stargardt disease patients in Portugal [19, 20]. Another showed an understanding of factors that may be hindering the registry’s nationwide adoption, given the lower-than-expected adoption rate [21]. A recent study based on the first nationwide survey in Portugal, yet unpublished, estimated the IRD prevalence of 0.031%, i.e., about 1 in 3000 individuals (Marques et al., submitted). This estimation was based on the Portuguese population and considering the survey’s broad national coverage. The electronic questionnaire was sent to 34 public healthcare providers and comprised some questions, including the number of IRD patients managed by each of them. However, there are still no studies on national epidemiology according to the regions of the country and the socioeconomic characteristics of the municipalities where these patients live. Knowing the target population and its resources is essential to better plan support measures.

The aim of this study was to evaluate the socioeconomic characteristics of regions in Portugal where IRD patients reside to allow for effective intervention measures.

## Results

### Demographic data

This study included 1082 patients from 973 families, 48.2% male and 51.8% female, aged 3 to 92 years, with a mean age of 44.8 ± 18.1 years, with 82 (7.6%) cases of paediatric age (under 18 years), 862 (79.7%) cases of working-age (between 15 and 64 years) and 164 (15.2%) elderly people (65 or more years).

The last Portugal population registry accounted for 10,344,802 inhabitants giving us a minimal prevalence of 1:9561 or 10.4 per 100,000 inhabitants. Considering a worldwide IRD prevalence of 1:1000-1:3000, our cohort is likely to represent 10.5–31.4% of the total patients with IRD in Portugal [22, 23]. Considering a national IRD prevalence of 1:3000 based on a recent unpublished study, our cohort is likely to represent 31.4% of the total patients with IRD in Portugal (Marques et al., submitted).

**Fig. 1:**
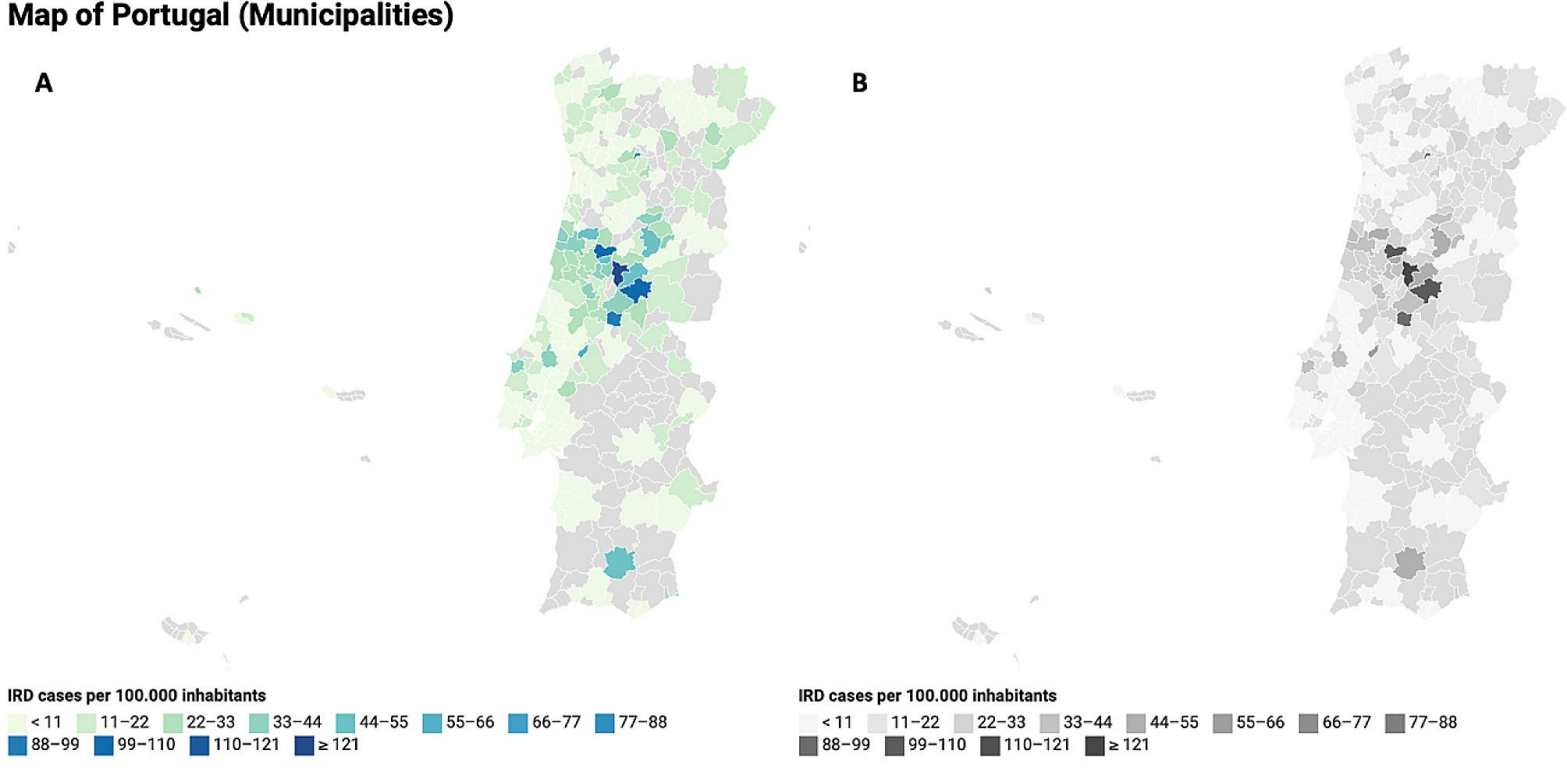
Regional distribution of cases and prevalence according to Municipalities. (**A**) coloured version. (**B**) accessible version for achromatopsia patients

Regional distribution of cases and prevalence according to NUTS I and NUTS II regions can be seen in Table 1 and according to Municipalities in Fig. 1A (coloured version) and 1B (accessible version for achromatopsia readers). Overall, IRD patients were identified living in 190 of the 308 municipalities. The prevalence by municipalities ranges from 0 to 131.2 per 100,000 inhabitants. The 75th percentile corresponded to 21.9 per 100,000 inhabitants (about 2.1x the national prevalence) and 47 municipalities are present at or above this percentile. The 90th percentile was 33.3 per 100,000 inhabitants (about 3.2x the national prevalence) and 20 municipalities are present at or above this percentile. Above or at the 95th percentile (46.0 per 100,000 inhabitants, 4.4x more than national prevalence), nine municipalities were identified (value per 100,000 inhabitants): Seia (46.0), Pampilhosa da Serra (49.0), Anadia (50.8), Golegã (55.6), Mesão Frio (84.6), Vila de Rei (91.5), Penacova (99.1), Oleiros (101.9) and Góis (131.2).

**Table 1:** Global characterization of the sample

Number of patients	1082
**Number of families**	973
**Age**	
Mean	44.8±18.1
Interval	3–92
**Gender**	
Male	522 (48.2%)
Female	560 (51.8%)
**Public Healthcare Providers**	
Centro Hospitalar Universitário de Coimbra (CHUC)*	396 (36.6%)
Instituto Oftalmológico Dr. Gama Pinto (IOGP)	264 (24.4%)
Centro Hospitalar Universitário de Santo António (CHUdSA)*	243 (22.5%)
Centro Hospitalar Universitário de Lisboa Norte (CHULN)*	69 (6.4%)
Hospital de Braga (HB)*	66 (6.1%)
Centro Hospitalar Lisboa Ocidental (CHLO)	44 (4.1%)
**Area of residence (per 100,000 inhabitants)**	
**NUTS I**	
Continental Portugal	10.8
Azores	4.7
Madeira	0.8
**NUTS II**	
Norte	9.7
Centro	16.7
Lisbon Metropolitan Area	9.7
Alentejo	7.4
Algarve	4.1
Azores	4.7
Madeira	0.8

*The only current users of the national web-based IRD registry (IRD-PT)

***Abbreviations***: *NUTS, Nomenclature of Territorial Units for Statistics.*

Non-syndromic (ORPHA 791) and syndromic retinitis pigmentosa (ORPHA 519,325) were the most frequent diagnoses, 44.8% and 14.1% respectively. Cone and cone-rod dystrophies (ORPHA 1872) and Stargardt disease (ORPHA 827) were the following most frequent diagnoses, with 12.6% and 8.3% of patients respectively. Distribution by clinical diagnoses including the respective ORPHA numbers can be seen in Table 2.

**Table 2:** Distribution of clinical diagnoses including the respective ORPHA numbers in the cohort

INHERITED RETINAL DYSTROPHIES (ORPHA 71,862)	NUMBER	%
**Isolated Progressive Inherited Retinal Disorder (ORPHA 519,306)**	795	73.5
Non-syndromic retinitis pigmentosa (ORPHA 791)	484	44.8
Cone and cone-rod dystrophy (ORPHA 1872)	136	12.6
Stargardt disease (ORPHA 827)	90	8.3
Leber congenital amaurosis (ORPHA 65)	44	4.1
Best vitelliform macular dystrophy (ORPHA 1243)	14	1.3
Pattern dystrophy (ORPHA 63,454)	12	1.1
Non-specific macular dystrophy	10	0.9
Retinitis punctata albescens (ORPHA 52,427)	4	0.4
Sorsby macular dystrophy (ORPHA 59,181)	1	0.1
**Syndromic Inherited Retinal Disorder (ORPHA 519,325)**	**163**	**15.1**
Syndromic retinitis pigmentosa (ORPHA 519,325)	152	14.1
Maternally-Inherited Diabetes and Deafness (ORPHA 225)	4	0.4
Pseudoxanthoma elasticum (ORPHA 758)	3	0.3
Hypotrichosis with juvenile macular dystrophy (ORPHA 1573)	2	0.2
Papillorenal syndrome (ORPHA 1475)	1	0.1
Sjogren-Larsson syndrome (ORPHA 816)	1	0.1
**Isolated Stationary Inherited Retinal Disorder (ORPHA 519,319)**	**40**	**3.7**
Achromatopsia (ORPHA 49,382)	19	1.8
Early-Onset “Drusenoid” Macular Dystrophies (ORPHA 75,376)	11	1.0
Fundus albipunctatus (ORPHA 227,796)	8	0.7
Congenital Stationary Night Blindness (ORPHA 215)	2	0.2
**Chorioretinal Dystrophies (ORPHA 519,300)**	**35**	**3.2**
Choroideremia (ORPHA 180)	14	1.3
Gyrate atrophy of choroid and retina (ORPHA 414)	7	0.6
Central areolar choroidal dystrophy (ORPHA 75,377)	6	0.6
Bietti crystalline retinopathy (ORPHA 41,751)	4	0.4
Non-specific chorioretinal dystrophy	4	0.4
**Inherited vitreous dystrophies (ORPHA 519,304)**	**27**	**2.5**
X-linked retinoschisis (ORPHA 792)	23	2.1
Enhanced S Cone Syndrome/Goldmann-Favre (ORPHA 53,540)	4	0.4
**Other Rare Disorders of the Posterior Segment of the Eye (ORPHA 519,311)**	**21**	**1.9**
Ocular and oculocutaneous (ORPHA 284,804 e 55) albinism	16	1.5
Foveal hypoplasia (ORPHA 519,398)	4	0.4
Posterior microphthalmos	1	0.1

*Bold corresponds to items (groups of diseases) that have dependences*

### Socioeconomic data

Table 3 shows all parameters organized by the main topics mentioned above and their values according to the national population (308 municipalities), the municipalities where no enrolled IRD patients reside (118 municipalities), the municipalities where enrolled IRD patients reside (190 municipalities) and the municipalities with a prevalence equal to or greater than the 75th (47 municipalities) and 90th (20 municipalities) percentiles.

**Table 3:** Socioeconomic parameters analysis according to the national population (308 municipalities), the municipalities where no IRD patients reside (118 municipalities), the municipalities where IRD patients reside (190 municipalities) and the municipalities with a prevalence equal to or greater than the 75th (47 municipalities) and 90th (20 municipalities) percentiles

	NAT Value 2021* (*N* = 308)	MUN without IRD (*N* = 118)	MUN with IRD (*N* = 190)	MUN with IRD PR ≥ P75 (*N* = 47)	MUN with IRD PR ≥ P90 (*N* = 20)
**POPULATION**					
**Population density** Number of people per km^2^	112.2	93.6 (±294.1)	416.4 (±975.6)	103.6 (±151.1)	67.2 (±47.9)
**Working age population rate** Between 15 and 64 years (%)	63.7	59.5 (±5.7)	61.4 (±4.6)	58.7 (±4.6)	57.7 (±4.7)
**Elderly population** Older than 65 years (%)	23.4	29.4 (±7.5)	26.8 (±6.4)	30.5 (±6.4)	32.1 (±6.6)
**Ageing index** Elderly per 100 young people	182.1	288.7 (±136.0)	244.1 (±114.9)	305.1 (±133.0)	346.0 (±161.8)
**Total dependency index** Young and old per 100 people of working age	57	69.6 (±16.5)	63.9 (±13.2)	71.4 (±14.1)	74.5 (±15.5)
**Resident foreign population rate** Foreign nationals in total population (%)	6.7	5.1 (±7.6)	4.3 (±4.4)	3.5 (±2.9)	3.3 (±2.1)
**Crude birth rate** Births per 1000 residents	7.7	6.4 (±2.0)	6.7 (±1.6)	6.0 (±1.4))	5.8 (±1.7)
**Crude death rate** Deaths per 1000 residents	12	17.4 (±5.4)	14.1 (±4.3)	16.5 (±4.5)	17.5 (±4.5)
**Child mortality rate** Children under one year old died per 1000 births	2.4	2.2 (±9.2)	2.1 (±5.7)	2.5 (±8.9)	1.6 (±5.5)
**EDUCATION**					
**Illiteracy rate** People who cannot read or write (%)	3.1	5.6 (±2.3)	4.2 (±1.9)	5.1 (±1.8)	5.2 (±2.0)
**No educational qualifications** Resident population aged 15 (%)	5.9	9.8 (±2.9)	7.4 (±2.6)	8.8 (±2.4)	9.0 (±2.6)
**1st cycle as the highest educational level obtained** Resident population aged 15 and over (%)	22.3	29.5 (±5.6)	26.7 (±6.3)	30.5 (±5.6)	31.4 (±5.6)
**2nd cycle as the highest educational level obtained** Resident population aged 15 and over (%)	9.6	11.1 (±2.2)	10.2 (±2.1)	9.9 (±1.6)	9.5 (±1.1)
**3rd cycle as the highest educational level obtained** Resident population aged 15 and over (%)	17.8	17.1 (±2.5)	17.5 (±2.0)	17.0 (±2.1)	17.1 (±2.3)
**Upper-secondary as the highest educational level obtained** Resident population aged 15 and over (%)	23.5	20.1 (±3.0)	22.0 (±3.3)	20.3 (±2.6)	20.4 (±2.8)
**Higher education as the highest educational level obtained** Resident population aged 15 and over (%)	19.8	11.5 (±2.7)	15.2 (±5.8)	12.6 (±4.6)	11.7 (±2.8)
**HEALTH**					
**Inhabitants per doctor** Average number of people per doctor	176.4	644.4 (±314.9)	438.7 (±292.4)	606.4 (±410.1)	689.5 (±498.6)
**Inhabitants per pharmacist** Average number of people per pharmacist	645.4	1253.7 (±531.3)	923.6 (±422.8)	1056.0 (±673.4)	937.8 (±343.8)
**Inhabitants per non-specialist doctor** Average number of people per	457.9	1252.1 (±809.8)	877.3 (±562.4)	1081.0 (±666.1)	1135.1 (±658.7)
**Inhabitants per general practitioner** Average number of people per general practitioner	1261.9	2745.6 (±1876.1)	2157.4 (±1534.6)	2496.2 (±1560.8)	2710.8 (±1429.7)
**EMPLOYMENT AND LABOUR MARKET**					
**Registered unemployed rate** People between 15 and 64 years looking for a job registered in the IEFP (%)	5.9	6.2 (±2.1)	5.5 (±2.0)	5.2 (±2.2)	4.7 (±1.9)
**SOCIAL PROTECTION**					
**Unemployment allowance of Social Security** Beneficiaries per 100 residents	1.6	1.4 (±0.6)	1.4 (±0.6)	1.1 (±0.4)	1.0 (±0.3)
**Guaranteed Minimum Income and Social Security Integration Benefit** Beneficiaries per 100 residents	2.9	3.4 (±2.8)	2.5 (±1.7)	2.2 (±1.4)	2.0 (±1.0)
**Disability allowance of Social Security** Beneficiaries per 100 residents	1.0	0.8 (±0,5)	1.0 (±0.4)	0.8 (±0.3)	0.7 (±0.3)
**Tertiary care allowance of Social Security** Beneficiaries per 100 residents	0.1	0.1 (±0.1)	0.1 (±0.0)	0.1 (±0.0)	0.1 (±0.1)
**Pension of Social Security (any type)** Beneficiaries per 100 residents	28.8	33.0 (±9.3)	31.0 (±6.3)	35.0 (±6.3)	36.9 (±6.4)
**Elderly pension of Social Security** Beneficiaries per 100 residents	20.0	22.3 (±5.8)	21.3 (±4.4)	23.8 (±4.4)	25.1 (±4.6)
**Disability pension of Social Security** Beneficiaries per 100 residents	1.7	2.2 (±0.9)	1.9 (±0.7)	2.1 (±0.8)	2.2 (±0.7)
**Survivor pension of Social Security** Beneficiaries per 100 residents	7.1	9.0 (±2.0)	7.9 (±1.8)	9.0 (±1.9)	9.6 (±1.9)
**HOUSING, COMFORT AND LIVING CONDITIONS**					
**Bank valuation per m** ^**2**^ **of houses** Median value in Euro	1231.0	945.97 (±346.1)	972.5 (±379.1)	786.8 (±196.0)	725.3 (±108.0)
**ENVIRONMENT, ENERGY AND TERRITORY**					
**Water quality for human consumption** Piped and good quality water (%)	99.0	98.9 (±1.7)	99.3 (±0.9)	99.2 (±0.8)	99.1 (±0.7)
**Street lighting** Electricity consumption per kilowatt-hour (kWh)	107.30	196.4 (±156.1)	138.5 (±73.7)	176.8 (±91.7)	194.5 (±105.8)
**Maximum altitude** Compared to mean sea level (m)	2351	768.6 (±491.1)	674.3 (±451.7)	759.3 (±495.5)	844.6 (±541.0)
**Minimum altitude** Compared to mean sea level (m)	0	105.5 (±126.4)	73.6 (±105.0)	100.8 (±110.4)	124.9 (±128.1)
**Rural fires** Fires in forests, bushes or farms by municipality	29.4	17.9 (±16.4)	35.6 (±34.9)	15.3 (±16.4)	10.3 (±7.2)
**JUSTICE AND SECURITY**					
**Total crimes** Registered by the police per 1000 inhabitants	29.1	27.7 (±10.7)	25.5 (±7.6)	23.0 (±6.8)	22.9 (±6.4)
**Domestic violence against a spouse or equivalent** Registered by the police per 1000 inhabitants	2.2	2.3 (±1.1)	2.1 (±0.7)	2.0 (±0.7)	2.2 (±0.9)
**ELECTORAL PARTICIPATION**					
**Abstention rate for Assembly of the Republic election** In 2022 (%)	48.6	47.2 (±8.1)	44.0 (±5.8)	44.2 (±4.7)	43.4 (±4.3)
**Abstention rate for Presidency of the Republic election** In 2021 (%)	60.8	59.5 (±6.3)	57.7 (±5.9)	59.1 (±5.4)	58.8 (±6.0)
**Abstention rate for Local Authorities election** In 2021 (%)	46.4	36.1 (±8.2)	42.1 (±8.7)	37.7 (±7.6)	36.4 (±7.6)
**Abstention rate for European Parliament election** In 2019 (%)	69.3	68.4 (±6.7)	67.1 (±5.4)	67.4 (±5.6)	66.3 (±6.2)
**CULTURE**					
**Spectators in movie theaters** Number per 1000 residents	528.9	64.4 (±279.1)	213.6 (±441.4)	71.0 (±228.5)	42.4 (±113.6)
**Spectators at live art events** Number per 1000 residents	344.4	328.6 (±274.2)	331.9 (±360.7)	462.6 (±543.9)	491.9 (±652.5)
**TOURISM**					
**Nights in tourist accommodations** Number per 100 inhabitants	360.3	590.1 (±976.3)	319.0 (±759.9)	335.3 (±627.2)	248.3 (±378.8)
**Foreign guests in tourist accommodations** Number per 100 inhabitants	40.9	21.7 (±20.0)	19.1 (±14.2)	13.5 (±9.7)	11.2 (±7.5)
Numeric values correspond to mean (± standard deviation) Abbreviations: IEFP, Institute of Employment and Training; IRD, inherited retinal dystrophy; MUN, municipalities; N, number; NAT, national; PR, prevalence					

#### Population

Overall, in regions with a higher prevalence of patients living with an IRD, the population is older (*r* = 0.475, *p* < 0.001), there is less working-age population (*r*=-0.453, *p* < 0.001), a higher ageing index (*r* = 0.470, *p* < 0.001), a higher total dependency index (*r* = 0.453, *p* < 0.001), a lower crude birth rate (*r*=-0.407, *p* < 0.001) and a higher crude death rate (*r* = 0.442, *p* < 0.001). Population density also decreased with the increase in IRD prevalence (*r*=-0.371, *p* < 0.001).

#### Education

The education status varied according to IRD prevalence. Municipalities with a higher IRD prevalence had a higher illiteracy rate (*r* = 0.404, *p* < 0.001), a higher proportion of people with no education qualifications (*r* = 0.416, *p* < 0.001) and a lower proportion of people with higher education (*r*=-0.391, *p* < 0.001).

#### Health

IRD patients have more difficult access to healthcare in their municipalities. The proportion of inhabitants per doctor is higher with increasing IRD prevalence (*r* = 0.350, *p* < 0.001).

The number of municipalities without ophthalmologists was 198 (64.3%) in Portugal, 98 (51.6%) in municipalities with IRD patients, 38 (80.8%) in municipalities with a prevalence greater or equal to the 75th percentiles and 18 (90.0%) in municipalities with a prevalence greater or equal to the 90th percentiles.

The number of municipalities without psychiatrists was 180 (58.4%) in Portugal, 85 (44.7%) in municipalities with IRD patients, 33 (70.2%) in municipalities with a prevalence greater or equal to the 75th percentiles and 15 (75.0%) in municipalities with a prevalence greater or equal to the 90th percentiles.

#### Employment and labour market

Although the registered unemployment rate seemed to be lower in municipalities with higher IRD prevalence, the correlation was not statistically significant (*r*=-0.130, *p* = 0.079).

#### Social protection

In municipalities with higher IRD prevalence, there are fewer beneficiaries of social security allowances: Unemployment (*r*=-0.469, *p* < 0.001), Guaranteed Minimum Income and Social Security Integration (*r*=-0.152, *p* = 0.036), Disability (*r*=-0.300, *p* < 0.001) and Tertiary care (*r*=-0.291, *p* < 0.001). The opposite trend happens with social security pensions, which are positively correlated with the prevalence of IRD: total (*r* = 0.439, *p* < 0.001), Elderly (*r* = 0.403, *p* < 0.001), Disability (*r* = 0.182, *p* = 0.012) and Survivorship (*r* = 0.476, *p* < 0.001).

#### Housing, comfort and living conditions

The median value of bank evaluation per m^2^ of houses decreased with the increase in IRD prevalence (*r*=-0.431, *p* < 0.001).

#### Environment, energy and territory

The water quality for human consumption decreased (*r*=-0.194, *p* = 0.008) and street lighting increased (*r* = 0.265, *p* < 0.001) with the increase in IRD prevalence. Regarding territory, municipalities with a higher IRD prevalence had a higher maximum altitude (*r* = 0.185, *p* = 0.011), a higher minimum altitude (*r* = 0.287, *p* < 0.001) and a smaller number of rural fires (*r*=-0.449, *p* < 0.001).

#### Justice and security

In municipalities with higher IRD prevalence, there were fewer crimes (*r*=-0.177, *p* = 0.015), and the same tendency to domestic violence (*r*=-0.059, *p* = 0.423) than in others.

#### Electoral participation

The abstention rate tendency is different according to the type of election. The abstention rate significantly correlated with the IRD prevalence: positively in the Presidency of the Republic election (*r* = 0.260, *p* < 0.001) and negatively in the Local Authorities election (*r*=-0.375, *p* < 0.001). No correlations were found between the abstention rate and IRD prevalence in the National Parliament election (*r* = 0.122, *p* = 0.092) and the European Parliament election (*r* = 0.141, *p* = 0.053).

#### Culture

The audience at the cinema is lower with the increase in IRD prevalence (*r*=-0.315, *p* < 0.001) and there was no correlation between the audience at live art events and IRD prevalence (*r* = 0.105, *p* = 0.240).

#### Tourism

Nights in tourist accommodations were not correlated with IRD prevalence (*r* = 0.021, *p* = 0.778), but the proportion of foreign guests in tourist accommodations was lower in municipalities with higher IRD prevalence (*r*=-0.287, *p* < 0.001).

#### Multivariate analysis

A multivariate analysis was performed in order to understand which of these socioeconomic factors was most influential on IRD prevalence. The model included socioeconomic factors without high collinearity between them (ageing index, no education qualifications, inhabitants per doctor, beneficiaries of social security unemployment allowance, bank evaluation per m^2^ of houses, street lighting, crimes, abstention rate in Local Authorities election, audience at the cinema, and foreign guests in tourist accommodations). The results are presented in Table 4. Adjusted R^2^ for the model was 13.8%. As shown in the model, “inhabitants per doctor” was the most important socioeconomic factor related to IRD prevalence.

**Table 4:** Multivariate linear regression analysis. Adjusted model for ageing index, no education qualifications, inhabitants per doctor, beneficiaries of social security unemployment allowance, bank evaluation per m^2^ of houses, street lighting, crimes, abstention rate in Local Authorities election, audience at the cinema, and foreign guests in tourist accommodations. Dependent variable: IRD prevalence. Adjusted R^2^ for the model was 13.8%. Abbreviation: IRD, inherited retinal dystrophy

	Unstandardized B coefficient	Standardized B coefficient	*t*-value	*p*-value	95% Confidence Interval for B	Correlation
Intercept	16.525		2.005	0.047	0.251 to 32.799	
Ageing index	0.030	0.200	1.677	0.096	-0.005 to 0.064	0.335
No education qualifications	-0.105	-0.028	-0.242	0.809	-1.374 to 1.074	0.279
Inhabitants per doctor	0.010	0.195	2.228	0.027	0.001 to 0.019	0.300
Beneficiaries of social security unemployment allowance	-3.443	-0.157	-1.752	0.082	-7.325 to 0.438	-0.321
Bank evaluation per m^2^ of houses	-0.002	-0.066	-0.535	0.593	-0.010 to 0.006	-0.298
Street lighting	-0.008	-0.038	-0.401	0.689	-0.049 to 0.032	0.174
Crimes	-0.097	-0.060	-0.648	0.518	-0.393 to 0.199	-0.201
Abstention rate in Local Authorities election	-0.028	-0.018	-0.191	0.849	-0.320 to 0.264	-0.246
Audience at the cinema	0.000	-0.015	-0.163	0.871	-0.005 to 0.004	-0.220
Foreign guests in tourist accommodations	0.046	0.052	0.534	0.594	-0.123 to 0.214	-0.219

## Discussion

This is the first study evaluating the socioeconomic status of the regions where Portuguese IRD patients reside. The estimated IRD prevalence varied according to the region. Knowing the target population is essential for developing public health and social measures.

Overall, this study found that IRD patients live in municipalities with aged populations, which may lead to less investment or support. However, policies financed by community funds that facilitate accessibility both in terms of mobility (such as sidewalks and traffic lights with audible warnings) and in the social integration of the elderly (such as adapted gymnastics, music, or computer classes) may also benefit patients with an IRD. Additionally, IRD patients reside in more isolated localities, as observed by a higher prevalence in areas with less population density and localized at higher maximum and minimum altitudes.

Regarding education, illiteracy increased and qualifications decreased with the increase of IRD prevalence. Probably, there will be fewer opportunities for different mechanisms of learning in these municipalities too, this should alert all schools with IRD patients to the possibility of adapted learning and its importance. People with a greater level of education will have more capacity to integrate the labour market, even when adaptations are required.

Health is one of the most important topics, given the target population and the multivariate analysis. IRD patients have more difficulty accessing healthcare where they live, and this problem is worse in municipalities with a higher prevalence. Access should be improved or guaranteed. It is also important to sensibilize doctors about these diseases and the need for regular follow-up for continuous visual rehabilitation, as well as psychological support, due to the progressive course of most IRD types [17]. 

Concerning employment, in the municipalities where IRD patients live there was not a high level of unemployment, meaning more chances of finding work adapted to their abilities. Despite this, some municipal or national support should be considered to facilitate this framework, since many patients, despite not being completely incapacitated for work, have major visual limitations that do not fit into the current legislated special regimes for accessing the labour market.

In the topic of social protection, municipalities with higher IRD prevalence had fewer beneficiaries of social security allowances and more social security pensions. This is in line with an elderly population and a population that is probably less informed, as it has a lower education level and is more isolated. The tertiary care allowance, as well as the disability allowance, should be more publicized and facilitated to this target population, by the corresponding entities, which are often unaware of these situations.

In this study, regarding the environment, we selected water quality for human consumption, street lighting and rural fires. The quality of water for human consumption is an essential indicator for assessing the level of development of a country and the well-being of its population, and in this study, it proved to be worse in areas with a higher prevalence of IRDs [24]. The street lighting parameter was strategically chosen because of the large proportion of patients with retinitis pigmentosa who usually have nyctalopia. Although in municipalities with a higher IRD prevalence, there is a higher consumption of energy for street lighting, this does not mean that it is of sufficient light intensity and adequate distribution. The importance of street lighting for the safety of all citizens must be reinforced in an era in which energy savings are given priority. Rural fires happen less frequently in areas with greater IRD prevalence, however, these patients should be identified preventively by civil authorities, especially in more isolated areas, since they have more difficulties travelling, many are unable to drive, and therefore require assistance in case of evacuation.

In addition to the lower number of rural fires, there is also less crime in the municipalities with the highest IRD prevalence, which is important for these patients, who may be more vulnerable.

The highest electoral involvement was mainly in elections for the Local Authorities, where there is a lower abstention rate in these areas of greater IRD prevalence. Together with local authorities, it is important to mobilize and make people aware of these pathologies. There is a lot of negative discrimination toward these patients since they are often young people with normal-looking eyes, without syndromic facies, but with blind behaviour that is misunderstood by many, leading to the accusation of simulation to obtain labour and social gains.

Finally, culture can be a channel for the dissemination of information about these diseases through advertising campaigns that are shown between artistic events, whether in the cinema or at live events. The revenue from the higher tourism in these regions could be channelled to provide a better transport network in the municipalities.

This is a pioneer study as it is the first to document these social and economic differences according to IRD prevalence using national statistics for this group of pathologies, but further research will be needed, including in other countries.

We excluded patients who died or do not live permanently in Portugal because the aim of this study was to characterize the environment where these patients reside, to understand possible interventions in the present.

One of the strengths of this study is its multicentric design, which included all IRD specialists who are current users of the national, web-based IRD registry (IRD-PT), aggregating a significant sample of patients nationwide, and its distribution. This allows us to know where there is a greater potential need for the creation of intervention measures. Another strength is the range of credible socioeconomic data made available in PORDATA allowing us to form a complete and comprehensive portrait of the municipalities where these patients live. The current data, based on CENSUS 2021, includes the socioeconomic changes caused by the covid-19 pandemic era.

However, this study has some limitations. The socioeconomic status of municipalities where IRD patients live was evaluated, not the patient’s socioeconomic status. For this reason, the visual aid strategies discussed were based on deduction and data should be interpreted with caution.

Another limitation is the missing data in some Portuguese municipalities. There were 38% (118/308) municipalities with no IRD patient identified. On the other hand, the average IRD prevalence captured by this study is about 1/10,000. This means, that this study should have identified at least one IRD patient in each of these 118 municipalities if the population of each of these municipalities exceeds 10,000. However, comparing municipalities with and without IRD patients identified, there were 19% (37/190) and 71% (84/118) municipalities with less than 10,000, respectively. In other words, municipalities without IRD patients identified had fewer inhabitants and were less likely to have any cases present.

Other additional reasons for missing data could related to the study method/design. Data from three out of nine IRD experts and two out of five tertiary hospitals were absent from this study. There is the possibility of some IRD patients from these 118 municipalities having more convenient access to the IRD experts and tertiary hospitals not participating in this study. Additionally, missing data could be explained by not all patients being referred to a tertiary hospital (although this is usually done in Portugal), not all patients with IRD having been identified, or representing actual clusters of these inherited diseases. Looking at Fig. 1, it appears that many of the municipalities with no identified IRD patients are in rural areas of the country (also supported by a great percentage of these municipalities having less than 10,000 inhabitants), which could mean a lower diagnosis rate in these areas due to less access to health care. The data in Table 3 also supports this, since municipalities with no IRD enrolled had a higher proportion of inhabitants per doctor. A free IRD survey or screening in those municipalities with no IRD patient identified could help better understand if a severe underdiagnosis is present or confirm their absence. This solution could have a big impact on the diagnosis and clinical management of the disease prognosis for IRD patients in these municipalities.

On the other hand, the centre of the country had more municipalities with higher prevalence. This can represent regions of higher consanguinity and disease awareness inside the communities as well as smaller distance to referral tertiary hospital. The next step of this working group will be the genetic analysis of this population. “Hotspots” due to a high rate of consanguinity/homozygosity may allow for an earlier diagnosis, further increasing the difference between municipalities [14, 16, 23]. However, this effect will tend to decrease in the younger population given the migration from the interior to the coast (from rural to urban environments) that occurred in Portugal a few decades ago. In the subsequent assessment, it will be important to analyse migration and emigration data that may modify the pool for consanguinity.

In the future, we hope that IRD-PT will have more users and that national investigations into these rare diseases will increase with the collaboration of other tertiary hospitals.

This study can be an example to be replicated in other countries. This design allows us to understand which socioeconomic inequalities are present in addition to the visual inequalities already known about these patients.

## Conclusions

The number of patients living with an Inherited retinal disease (IRD) varied between Portuguese regions. Using data from national statistics (PORDATA), we observed differences in socioeconomic characteristics between regions. Multiple targeted vision aid and rehabilitation strategies can be developed to ensure that all IRD patients are granted full clinical and socioeconomic support.

## Methods

### Study design

**Fig. 2:**
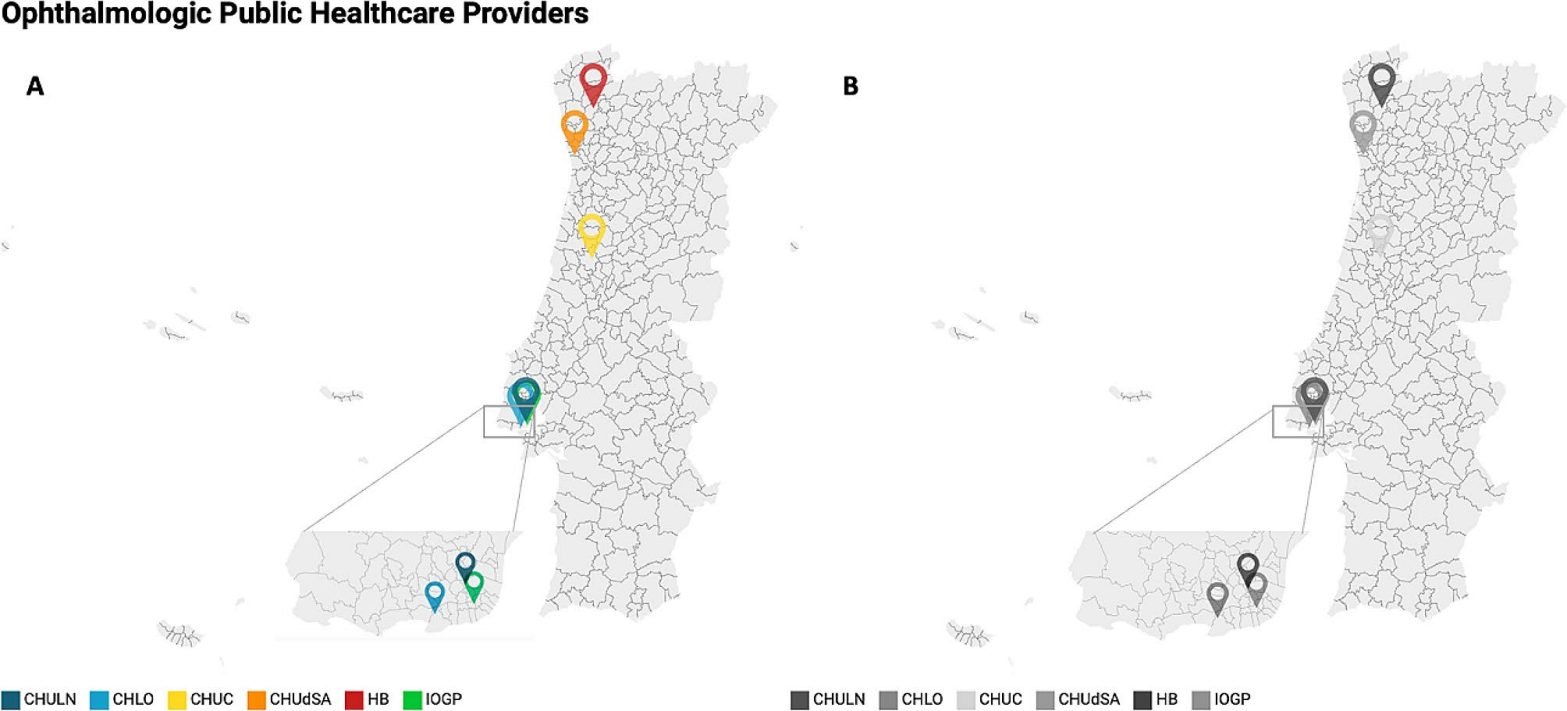
Location of participating public healthcare providers. (**A**) coloured version. (**B**) accessible version for achromatopsia patients

A multicentre, cross-sectional, cohort study of consecutive patients with a clinical diagnosis of IRD, identified from all three NUTS I regions (Nomenclature of Territorial Units for Statistics). The invitation to participate in this study was directed to all national IRD experts among public healthcare providers (HCPs) who receive and follow these patients. A response to the invitation to collaborate in this study was obtained from six IRD experts (66.7% response rate) from six HCPs from the three most populous (Norte, Centro and metropolitan Lisbon area) NUTS II regions. The regional distribution of participating HCPs can be seen in Fig. 2A (coloured version) and 2B (accessible version for achromatopsia readers). Three of the participating HCPs (Centro Hospitalar Universitário de Santo António, Centro Hospitalar e Universitário de Coimbra and Centro Hospitalar Universitário de Lisboa Norte) are tertiary hospitals (from a total of 5 nationwide) [25]. All current users of the national web-based IRD registry (IRD-PT) participated in the present study (correspond to four of the six centres included and are identified in Table 1).

Data was collected between October 2022 and January 2023 by each IRD specialist and their collaborators. This period allowed each expert to organize, code and share their own database, including all patients followed regularly in each HCP, and not only those who were observed in that period. The authors ensured that all patients’ anonymity was carefully protected and the study complied with the tenets of the Declaration of Helsinki for biomedical research.

### Participants

The inclusion criteria were: clinical diagnosis of IRD, known or ongoing genetic testing results and residence in Portugal.

The exclusion criteria were: death and emigration. Additionally, given the impossibility of identifying individuals by anonymized data sent from each HCP, cases with the same date of birth, residing in the same municipality, with the same clinical and genetic diagnosis, without a family history, and with follow-up at different hospitals, were considered repeated and deleted from this sample.

### Parameters

This study analysed:

- demographic characteristics of the study’s population (gender, age on 1st January 2023, and the first 4 digits of the residence’s postal code);
- clinical features [clinical diagnosis based on classification covered by the national, web-based IRD registry (IRD-PT) [18]; family history; genetic testing];
- socioeconomic environment of municipalities where each IDR patient lives, estimated by public statistical data.

Patients’ zip codes were converted to Eurostat NUTS levels. Socioeconomic variables of each region were collected from the “PORDATA– Statistics about Portugal and Europe” website, using the latest data from the 2021 CENSUS [26]. The main topics explored were: population; education; health; employment and labour market; social protection; housing, comfort and living conditions; environment, energy and territory; justice and security; electoral participation; culture; and tourism.

### Statistical analysis

Statistical analysis was performed using the SPSS program (IBM SPSS Statistics, version 28.0.1.0 for Mac). The normality of the variables was evaluated by the Kolmogorov-Smirnov test. Spearman’s bivariate correlation test was used to study linear correlations. For interpretation, a correlation coefficient was considered “very weak” if between 0 and ± 0.19, “weak” if between ± 0.20 and ± 0.39, “moderate” if between ± 0.40 and ± 0.59, “strong” if between ± 0.60 and ± 0.79 and “very strong” if between ± 0.80 and ± 1.0. Multivariate linear regression analysis, using generalized linear models adjusted for socioeconomic factors was performed to assess the influence of them on the IRD prevalence. Results were expressed as unstandardized and standardized B coefficients. Variables with high collinearity were not included in the model. A *p*-value lower than 0.05 was considered statistically significant. Figures 1 and 2 were made with the tool datawrapper (© 2023 Datawrapper developed by Datawrapper GmbH and available on the website datawrapper.de) [27]. 

## Data Availability

The data that support the findings of this study are not openly available due to reasons of sensitivity and are available from the corresponding author upon reasonable request. Data are located in controlled access data storage at CHUdSA.

## Abbreviations

HCPs: Public Healthcare Providers
IRD: Inherited Retinal Diseases
NUTs: Nomenclature of Territorial Units for Statistics
